# Influence of Temperatures on Physicochemical Properties and Structural Features of Tamarind Seed Polysaccharide

**DOI:** 10.3390/molecules29112622

**Published:** 2024-06-03

**Authors:** Yantao Liu, Yujia Sun, Diming Li, Pengfei Li, Nan Yang, Liang He, Katsuyoshi Nishinari

**Affiliations:** 1 Glyn O. Phillips Hydrocolloid Research Centre, School of Life and Health Sciences, Hubei University of Technology, Wuhan 430068, China; liuyt0321@163.com (Y.L.); sunsy188@163.com (Y.S.); m17764105202@163.com (D.L.); katsuyoshi.nishinari@gmail.com (K.N.); 2 Food Hydrocolloid International Science and Technology Cooperation Base of Hubei Province, Hubei University of Technology, Wuhan 430068, China; 3 Key Laboratory of Chemistry and Engineering of Forest Products, State Ethnic Affairs Commission, Guangxi Key Laboratory of Chemistry and Engineering of Forest Products, Guangxi Minzu University, Nanning 530006, China; lipfgxun@126.com; 4 Key Laboratory of Biological and Chemical Utilization of Zhejiang Forest Resources, Department of Forest Foods, Zhejiang Academy of Forestry, Hangzhou 310023, China; lianghezjfa@hotmail.com

**Keywords:** tamarind seed polysaccharide, potato starch, structural features, dispersion mechanisms

## Abstract

Due to the high content of impurities such as proteins in tamarind seed polysaccharide (TSP), they must be separated and purified before it can be used. TSP can disperse in cold water, but a solution can only be obtained by heating the mixture. Therefore, it is important to understand the dispersion and dissolution process of TSP at different temperatures to expand the application of TSP. In this study, pasting behavior and rheological properties as a function of temperature were characterized in comparison with potato starch (PS), and their relationship with TSP molecular features and microstructure was revealed. Pasting behavior showed that TSP had higher peak viscosity and stronger thermal stability than PS. Rheological properties exhibited that *G*′ and *G*′′ of TSP gradually increased with the increase in temperature, without exhibiting typical starch gelatinization behavior. The crystalline or amorphous structure of TSP and starch was disrupted under different temperature treatment conditions. The SEM results show that TSP particles directly transformed into fragments with the temperature increase, while PS granules first expanded and then broken down into fragments. Therefore, TSP and PS underwent different dispersion mechanisms during the dissolution process: As the temperature gradually increased, TSP possibly underwent a straightforward dispersion and was then dissolved in aqueous solution, while PS granules initially expanded, followed by disintegration and dispersion.

## 1. Introduction

Tamarind seed polysaccharide (TSP), also known as xyloglucan (XG), is the primary component of tamarind seed kernels, which is an underutilized byproduct of the tamarind pulp industry [1]. The chemical structure of XG is made up of a (1→4) -β-D-glucan backbone that is partially substituted with α-D-xylopyranose at the O-6 position. Certain xylose residues have (1→2) β-D-galactosyl units substituted at the O-2 position [2]. TSP and other XG have been called “amyloid” because they have similar properties to starch, which becomes blue after being treated with an iodine/potassium iodide solution [3,4]. TSP is also known as “aging-free starch” because it has stronger stability without showing retrogradation like starch [5,6]. Additionally, TSP has excellent viscoelastic and thermal stability. It is categorized as a dietary fiber because of its high molecular weight and distinct chemical structure, which prevents its potential for digestion and hydrolysis by salivary amylase and other human digestive enzymes [6,7].

XG is composed of side chains that are either xylose or galactose–xylose, and a main chain that is similar to cellulose [8]. The side chain has a major impact on rheological properties, water solubility, and other biological functions [6]. Furthermore, in an aqueous solution, XG exhibits a tendency to self-associate [2]. The solubility, viscosity, and gelation of XG in water are significantly influenced by the variation in the substitution degree of xylose and/or galactose [9]. The xylose units are revealed to be more hydrophilic than glucose units [2]. The presence of side chains interferes with the effective hydrogen bonding between the main chains, making XG more easily soluble in water. Therefore, TSP exhibits a certain degree of water solubility, but the entire macromolecule cannot be completely hydrated. TSP has a high protein content (13–20%), and it is necessary to separate and purify it before use [2]. TSP is also considered a highly hydrophilic gum due to its rich side chains. They can be immediately dispersed even in cold water, but only when heated at higher temperature can they form a uniform solution, typically reaching their maximum viscosity 20–30 min after boiling [2,10]. Therefore, it is important to study the dissolution process of TSP before separation and purification, and it is also helpful for us to understand the changes in the structure and physicochemical properties of TSP.

The molecular structure of TSP has already been characterized extensively. TSP is mainly composed of xyloglucan, and the study of its fine structure is mainly carried out through enzymatic hydrolysis and partial acid hydrolysis to separate the oligosaccharide fragments obtained by using gas chromatography–mass spectrometry (GC–MS), matrix-assisted laser adsorption ionization–time-of-flight (MALDI-TOF) mass spectrometry, gas chromatography (GC), 1D/2D NMR spectroscopy, and Dionex high-pH anion exchange–high-performance liquid chromatography (HPAE–HPLC). For example, York et al. [11] used β-galactosidase digestion and spectroscopy to analyze the structure of oligosaccharides obtained from enzymatic hydrolysis of glucanase. The previously proposed structure, which was based on the analysis of isomeric octasaccharide mixes, was validated by the spectrum characterization of oligosaccharide alditols that were generated by borohydride reduction of this octasaccharide. Marry et al. [12] isolated several oligosaccharide fragments from purified TSP through enzymatic digestion and partial acid hydrolysis, ranging from two to nine consecutive residues. Moreover, two major types of oligosaccharides were obtained through commercial D-glucose enzymatic hydrolysis. The first group included oligomers of DP 7, 8, and 9, whereas the second group had a variety of oligomers of DP 12 to 18, according to HPAE-PAD liquid chromatography analysis. The complicated and random distribution of side chains in TSP is reflected in the diversity of isolated oligomers [13]. With a ratio of 13:39:48, xyloglucan is mostly made up of four different forms of oligosaccharides that repeat: one hepta-, two octa-, and one nona-saccharide [2].

The dissolution of polysaccharides in water is mainly influenced by temperature, their own structure, particle size and shape, etc. The most typical example is starch gelatinization [14]. There are two distinct stages of gelatinization of starch. At 20–60 °C, starch molecules bind to water in the first stage. At that moment, hydrogen bonds and van der Waals forces play an important role in maintaining the molecular structure of starch molecules. In the second step, hydrogen bonds between starch molecules begin to break, and water molecules combine with the hydroxyl groups of starch molecules when the temperature increases to 60–90 °C. Subsequently, starch gradually gelatinizes and dissolves in water. This finally causes crystal disintegration and damage to starch granules. Therefore, by comparing with the starch gelatinization process, it is possible to have a clear understanding of the dynamic changes in the structural and physicochemical properties of TSP under different treatment temperatures.

There is still a lack of a clear explanation for the dynamic changes in the structural and physicochemical characteristics of TSP at different treatment temperatures. It is particularly important to study the changes in structure and properties of natural and pretreated TSP at different temperatures. In this work, the pasting, dispersion, and structural features of TSP were studied by heating them at different treatment temperatures (50, 60, 70, and 90 °C) and compared with potato starch (PS). The purpose of this study is to reveal the pasting, thermal, and structural properties of TSP so as to provide useful information for the production and application of TSP as a thickener in the food industry.

## 2. Results

### 2.1. Composition of TSP

The monosaccharide composition of natural TSP was determined by HPAEC-PAD, and it was found that glucose (Glc) was 52.1%, xylose (Xyl) was 29.7%, and galactose (Gal) was 18.2%, with a monosaccharide ratio of 2.86:1.63:1 for Glc:Xyl:Gal. This result was slightly higher than prior reports in Gal and Xyl content in a molar ratio of 3.1:1.7:1 [15] but lower than the literature-reported molar ratio of 2.01:1.33:1 for Xyl and Gal contents [16]. Moreover, the monosaccharide ratios of TSP treated at different temperatures are shown in Table 1. As the temperature increased, the monosaccharides ratios of TSP at different treatment temperatures showed an increasing trend in both the glucose and the xylose content and a decreasing trend in the galactose content. More importantly, the total percentage of side chains (Xyl+Gal) in TSP gradually decreased with the increase in treatment temperatures. This indicates that TSP with more side chains (higher content of xylose and galactose) was more soluble under lower-temperature conditions, while TSP with a higher glucose percentage needed to be dissolved under higher-temperature conditions, which is consistent with previous reports [17].

As shown in Table 1, molecular weight parameters were determined for the TSP treated at different temperatures, and the elution profiles of TSP are shown in Figure S1. The values of *M*_w_ were slightly decreased from 9.622 × 10^5^ g/mol of TSP-50 to 8.584 × 10^5^ g/mol of TSP-90. The polydispersity indexes (*M*_w_*/M*_n_) were all not higher than 1.055, indicating that the TSP sample used in this study had extremely low polydispersity. The slight decrease in *M_w_* values indicates that TSP could withstand high temperatures while retaining structural stability.

### 2.2. Pasting Characteristics

**Figure 1:**
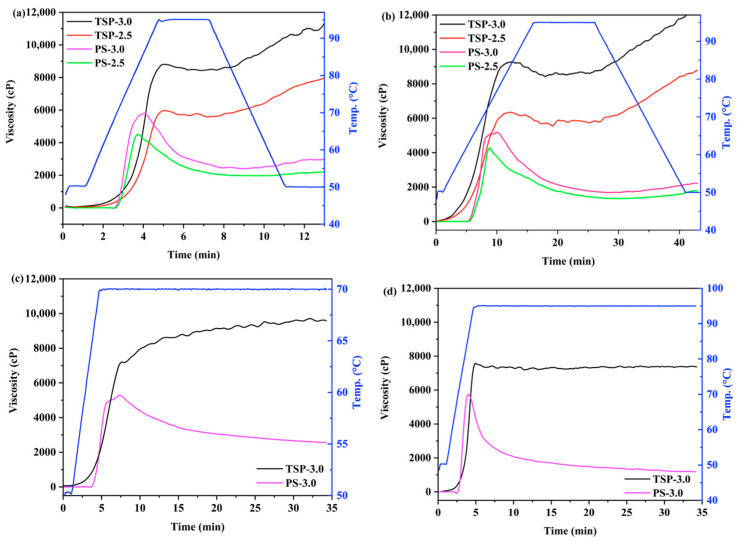
Pasting properties of TSP and PS: viscosity change during the pasting process following Standard 1 (**a**) and Standard 2 (**b**), and the viscosity changes in TSP and PS heated to 70 °C (**c**) and 95 °C (**d**), respectively, with a shearing rate of 160 r/min. The blue curve in each sub-figure represents the temperature profile for reference.

The viscosity profile of starch and other non-starch polysaccharides is one of the key indicators for evaluating their use as food raw materials or thickeners in the food industry and others [1]. The pasting properties of native TSP and PS suspensions with different concentrations determined by RVA are presented in Figure 1, and the pasting parameters are summarized in Table 2 (Standard 1) and Table S1 (Standard 2). The differences between Standard 1 and Standard 2 are the heating rate, holding time, and cooling rate during the thermal treatment. It was shown that the PS suspensions, under the treatment following Standard 1, exhibited traditional pasting profiles with variations in their pasting behaviors among different samples (Figure 1a); that is, the viscosity first remained unchanged, followed by an increased from PT to the PV, and then a decrease to MV before finally reaching its FV. In addition, it was found that the pasting temperature (PT) of PS-2.5 and PS-3.0, which are the temperatures at which the viscosity of the sample starts to increase after heating, were found to be approximately 69.90 °C and 69.43 °C, respectively, consistent with the earlier work by [18]. PS was by far the most viscous starch of all and had the lowest temperature of gelatinization [19]. On the contrary, compared with starch gelatinization curves, the viscosity of TSP significantly increased from the beginning of heating and did not exhibit typical PT. Then, it slightly decreased during the subsequent heating process and gradually increased during the following cooling process. For the peak viscosity (PV), shown in Figure 1a,b, TSP has much higher PV than PS at equal concentration when heat-treated with the same standard, and the peak time of TSP was also longer. Furthermore, TSP-2.5, a TSP sample with low concentration, had a PV of 6038 ± 46 cP, which was higher than the values of 5933 ± 193 cP for PS-3.0, a PS sample with a higher concentration (Table 2). In addition, we also found an interesting result that, unlike starch, the FVs (7995 cP and 11,227 cP) of TSP-2.5 and TSP-3.0 were much higher than their PVs (6038 cP and 8922 cP). On the one hand, the results indicate that it might have been caused by temperature effects. On the other hand, it could be indicated that TSP continued to dissolve or disperse into the aqueous solution from the start of heating; thus, the FVs after cooling could be still increased and be much higher than the PVs. The results also indicate that, compared to the thickening effect of PS, less TSP was needed as a thickening agent to achieve the same thickening effect in the food industry. The breakdown (BD, which refers to the difference between PV and MV) of starch paste could be used to characterize its heat and shear resistance [18]. As shown in Table 2, PS-2.5 and PS-3.0 had BD values of 2687 ± 31 cP and 3576 ± 190 cP, respectively. However, the BD values of TSP-2.5 and TSP-3.0 were only 567 ± 36 cP and 639 ± 151 cP, respectively, obviously lower than those of PS. The lower BD values of TSP might indicate that it was more resistant to high temperature and shearing during the heating process than PS. Previous research found that PS with a pasting characteristic of high PV and low BD was able to be used as a thickener or gelling agent in the food industry [20]. TSP, on the other hand, had significantly higher setback values (SB, which refers to the difference between FV and MV) than PS. The final viscosity (FV) of TSP-2.5 and TSP-3.0 was 7995 ± 237 cP and 11,227 ± 751 cP, respectively, which was 3.6 and 3.8 times that of PS-2.5 and PS-3.0. Therefore, the viscosity of TSP was obviously higher than that of PS dispersions of equal concentration, which is consistent with the results reported in previous studies [10,21].

Standard 1 is a general procedure that is often used for the pasting behavior of starch. Due to the relatively poor solubility of TSP in cold water, Standard 2 with a longer testing time might be able to ensure that TSP is completely dispersed and fully dissolved in aqueous solution. As shown in Figure 1b, the pasting behavior of PS in Standard 2 was similar to that of Standard 1. However, the PTs of PS-2.5 and PS-3.0 were reduced to 64.13 °C and 63.73 °C compared with those obtained from Standard 1, respectively. This might have been due to the slower heating rate of Standard 2, leading to a longer heating time for starch granules to swell and increase the viscosity. Similarly, the PV determined in Standard 2 for PS-2.5 and PS-3.0 was also lower than that determined in Standard 1. The results indicate that the PT of starch was significantly affected by the heating rate, as has been observed previously by other authors [22], while TSP-2.5 and TSP-3.0 had higher PVs than those determined in Standard 1, having increased by 6.5% and 6.3%, respectively. Compared to Standard 1, the FVs of PS-2.5 and PS-3.0 were lower. On the contrary, the FVs of TSP-2.5 and TSP-3.0 were higher than those in Standard 1. The different pasting behaviors of TSP and PS from both Standard 1 and Standard 2 might indicated that TSP and PS had different high-temperature stability and different dispersion mechanisms, and this will be further discussed in combination with rheological behavior and microstructure.

Because starch, as food additive, sometimes needs to undergo high temperatures or prolonged heating, it is necessary to conduct thermal stability tests on starch. In order to further compare the stability of TSP and PS suspensions at high temperature, as shown in Figure 1c,d, the viscosity curves at two selected temperatures (70 °C and 95 °C, one of which is near the PT of PS, and the other near the temperature of water boiling (cooking temperature of starch in daily life), respectively) were tested. At 70 °C, the viscosity of the PS suspension gradually increased and then decreased, while the TSP suspension kept increasing in viscosity during the whole heating process. However, at 95 °C, PS quickly reached its peak viscosity and then sharply decreased in viscosity, while the viscosity of TSP decreased a little after reaching the maximum and kept its high viscosity almost unchanged in the subsequent heating process. These results indicate that, unlike PS suspension, TSP had excellent stability against temperature. In addition to the temperature effect, it was found in a previous study that vigorous stirring could also reduce the viscosity of PS but had little effect on TSP [10]. Therefore, it is speculated that TSP could be used in a wide range of temperatures and mechanical processing conditions in the food industry with better performance than PS.

### 2.3. Rheological Properties

**Figure 2:**
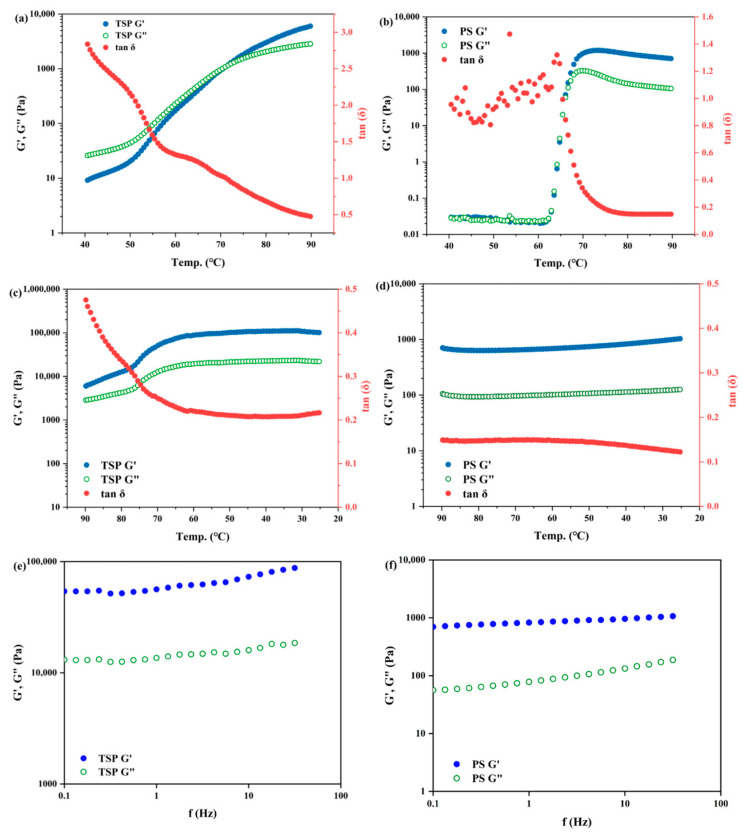
The dynamic oscillatory properties of TSP and PS. (**a**,**b**) *G*′, *G*″, and tan δ during temperature ramp from 40 to 90 °C; (**c**,**d**) *G*′, *G*″, and tan δ during temperature ramp from 90 to 25 °C; (**e**,**f**) *G*′ and *G*″ during a frequency sweep from 0.1 to 40 Hz.

In order to monitor the dynamic viscoelastic properties and evaluate the gelation performances under heating and non-heating situations, the dynamic rheology of TSP and PS was determined. Before conducting the rheological test, the TSP and PS samples were magnetically stirred under ambient temperature for half an hour (25 °C) to make sure they had dispersed uniformly and completely. For TSP and PS, respectively, temperature sweeps were first carried out, which included temperature increases during heating and temperature decreases while cooling. As depicted in Figure 2a, the *G*′ and *G*″ of TSP gradually increased with the increase in temperature during the heating process. *G*″ was higher than *G*′ at the beginning of heating; then, the intersection point appeared at around 71.0 °C, followed by *G*′ being higher than *G*″. The intersection of *G*′ and *G*″ might indicate that the weak network formed only after heating, as weakening of the bonds by heating allowed for the exchange of a sparsely cross-linked percolating network [2]. When the temperature continued to increase, *G*′ exceeded *G*″ and the dispersion exhibited elastic characteristics. As shown in Figure 2b, *G*′ and *G*″ of PS remained almost constant before reaching the onset temperature of gelatinization (about 64.3 °C). As the temperature increased from 64.3 °C to 73.5 °C, the *G*′ and *G*″ of PS increased sharply and achieved a maximum. A slow decrease was found in the *G*′ and *G*″ of PS when the temperature was further increased. The *G*′ and *G*″ of TSP increasing during the whole heating process might indicate that the dispersion mode of TSP particles was gradual in aqueous solution. On the other hand, it is known that PS granules usually absorbed water first, and then gradually swelled during the heating process in the presence of excess water. At the same time, amylose leached out from starch granules, and the starch molecular chains interacted with each other to form a network structure, ultimately leading to a rapid increase in the modulus of PS paste [23]. Moreover, it is hypothesized that the modest drop in *G*′ and *G*″ throughout the subsequent heating process could have been caused by the disintegration of starch granules and the weakening of interchain interactions [24]. The *G*′ and *G*″ values of TSP increased throughout the heating process and were much greater than those of PS, indicating that TSP exhibited good viscoelasticity behavior and high-temperature resistance characteristics. The results of the rheology are consistent with those of RVA. This might be due to the disintegration of PS particles at higher temperatures, while TSP could maintain structural stability under high temperature. As shown in Figure 2c,d, *G*′ was always higher than *G*″ for both TSP and PS as temperature decreased during the cooling process, which indicates that both TSP and PS had viscoelastic properties but that elasticity was dominant during the cooling process. However, in contrast with PS or other starches, the TSP dispersions did not form a gel after cooling. Therefore, TSP could be easily diluted and dispersed as a thickener even after storage for several days. However, the *G*′ and *G*″ of PS did not change significantly during the cooling process, whereas the *G*′ and *G*″ of TSP gradually increased with decreasing temperature, which was probably due to the enhancement of hydrogen bonding formation. To further confirm the dispersion mechanism of TSP, we recently conducted subsequent experiments: heating the TSP dispersion in a beaker to 90 °C and then decreasing it to room temperature, which is considered the first heating cycle. In the second heating cycle, we conducted it on a rheometer Mars 60, and the results are shown in the Supplementary Material (Figure S2). The TSP dispersion did not show the results exhibited in Figure 2. But as the temperature increased, the modulus gradually decreases, and then increases again with the increase of temperature, which indicated that TSP has dissolved in water after the first heating and cooling cycle.

In oscillation testing, Tan δ can be used to determine the onset of gelatinization by measuring the temperature at the maximum point [25]. As shown in Figure 2a, the Tan *δ* of TSP gradually decreased during the heating process, and the final value reached 0.47. The Tan *δ* of PS (Figure 2b) increased with the increase in temperature, reaching its maximum at 64.3 °C and then rapidly decreasing to 0.15, indicating the formation of an elastic gel. During the cooling process (Figure 2c,d), the Tan δ (less than 0.5) of TSP and PS gradually decreased overall, which indicates that their elastic properties improved with the decrease in temperature.

Additionally, frequency sweep tests were performed on the PS and TSP. As shown in Figure 2e,f, for both TSP and PS, *G*′ was greater than *G*′′ within the frequency range during testing, suggesting that both TSP and PS were more elastic than viscous. The *G*′ of PS was much higher and did not show strong frequency dependence as compared to the *G*″ of PS, indicating that a stable gel was formed. This stable gel structure was created by interactions between leached starch molecules in the continuous phase [24]. The *G*′ and *G*″ of TSP, on the other hand, exhibited a stronger frequency dependence than PS, which could have been due to TSP pastes did not form a stable gel and even easily redispersed in water.

### 2.4. Morphology and Microstructure of TSP and PS at Different Heating Temperatures

#### 2.4.1. Morphology of TSP and PS

**Figure 3:**
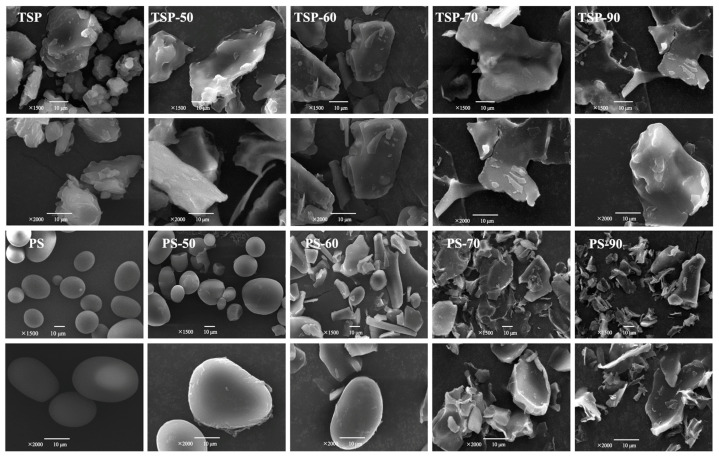
SEM images of TSP (1500×, 2000×) and PS (1000×, 2000×) at different temperatures. The number in the name of each sample indicates that the sample was not heated or heated at 50, 60, 70, or 90 °C.

The morphology of native TSP powders, PS granules, and these heat-treated samples was imaged and analyzed using SEM, which could visually observe the dynamic changes in their particle morphology at different temperatures. The morphology of untreated TSP powders, as depicted in Figure 3, revealed an intact appearance structure with irregular lump shape and various size, which was obviously different from the smooth but ellipsoid-shaped starch granules. The results indicate that the surface of natural PS granules appeared smooth, oval shaped, and free of cracks, which is consistent with previous studies on PS [26]. When the heat treatment temperature was 50 °C, the TSP particles became smooth, with sheet-like morphology. Then as the heat treatment increased to 70 °C and 90 °C, the TSP flakes became thinner, but the size of the TSP flakes did not change much. On the contrary, as shown in Figure 3, the morphology of PS granules underwent significant changes after the hydrothermal treatment. That is, PS-50 granules slowly expanded, leading to a slight increase in particle size but without destroying the granule structure. PS-60 granules continued to expand, causing some breakage, and the disruption of the PS-70 starch granules became aggravated as almost all starch granules broke, with no complete with starch granules seen in the field of vision. Finally, the PS-90 granules were further broken down into small fragments. These changes in PS granule morphology that resulted from hydrothermal treatment can be explained by the partial gelatinization of starch granules [27]. Initially, at low treatment temperature (50~60 °C), starch granules swell gradually until they are fully expanded near the gelatinization temperature (70 °C). During this process, the crystals of starch granules gradually disintegrate, and the swollen starch molecules interact with water to form a large number of hydrogen bonds. Subsequently, when the heating temperature increases above the point of starch granules, some hydrogen bonds between starch molecules break, and the hydroxyl groups of water molecules and the starch molecules begin to bind, disrupting the structure of the starch granules [28]. At this point, the double-helix structure of amylopectin gradually dissociates and is destroyed under continuous heating, and fragmentation of the swelling starch follows [28,29]. The changes in the viscosity, short-range ordered structure, solubility, and swelling property of starch granule are mainly caused by changes in their size, shape, and structure [30]. Hence, by comparing the SEM results, it is indicated that the dispersion or dissolution process of TSP and PS at different temperatures might have been influenced by their particle morphology.

#### 2.4.2. FT-IR Characterization of TSP and PS

**Figure 4:**
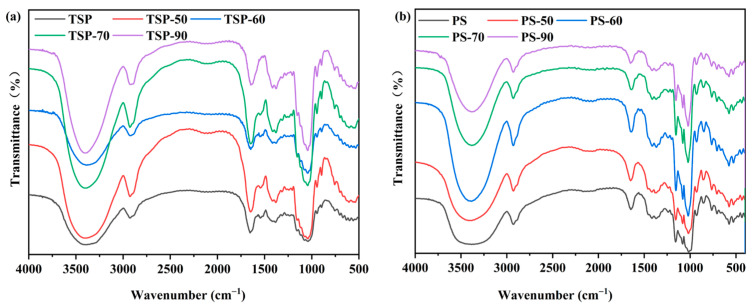
FT-IR spectra of TSP (**a**) and PS (**b**). The number in the name of each sample indicates that the sample was not heated or heated at 50, 60, 70, or 90 °C.

FT-IR analysis is traditionally used to evaluate the appearance, type, and strength of hydrogen bonds, which signifies alterations in the structure of polysaccharides, especially starch. The spectra of the TSP samples revealed all of the typical bands and peaks connected to polysaccharides, as seen in Figure 4a. A broad band at around 3401 cm^−1^ was discovered as a polysaccharide feature and was attributed to O-H stretching vibration generated by intra- or intermolecular forces [31]. An intense peak at 1645 cm^−1^ was ascribed to amide I (-CONH_2_ groups) and corresponded to the N-H bend of primary amines. The 1153 cm^−1^ and 1078 cm^−1^ bands, which are representative of carbohydrates, were found to contain pyranose. The 946 cm^−1^ band was created by xyloglucan’s ring vibration, while the 895 cm^−1^ band was caused by the C-H stretching characteristic of glucose and xylose β-anomeric linkages [32]. Spectra of native and four different-temperature-treated TSP samples showed a typical infrared spectrum for xyloglucan, as described in previous studies [32,33].

The spectra of native and different-temperature-treated PS are presented in Figure 4b. The stretching vibration of the O-H groups was characterized by a broad band at approximately 3385 cm^−1^. Furthermore, when the treatment temperature increased, the peak width at 3340 cm^−1^ shrank, suggesting that the hydrogen bond in PS weakened. The FT-IR spectra affected the starch granules’ short-range order [27]. The crystalline and amorphous portions of the starch are represented by the absorbance bands in the infrared spectrum at 1047 cm^−1^ and 1022 cm^−1^, respectively. In fact, the degree of organized structure in the starch was determined using the ratio 1047 cm^−1^/1022 cm^−1^. The FT-IR ratios of native PS, PS-50, PS-60, PS-70, and PS-90 were 1.012, 0.891, 0.773, 0.657, and 0.514, respectively. The ratios gradually decreased with the increase in processing temperature, which might have been due to the dissociation and decomposition of the double helix in the crystal region of starch molecules [27]. All of the peaks in Figure 4 appeared without any significant shifting for both TSP and PS, indicating that the hydrothermal treatment had no effect on their chemical structures.

#### 2.4.3. Crystalline Structure of TSP and PS

**Figure 5:**
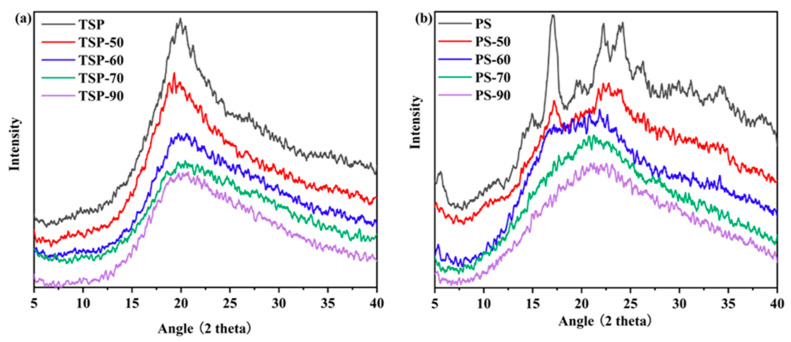
XRD analysis of TSP (**a**) and PS (**b**). The number in the name of each sample indicates that the sample was not heated or heated at 50, 60, 70, or 90 °C.

The XRD patterns of native TSP and PS, as well as the samples treated at different temperatures, were examined to determine whether they were amorphous or crystalline. All TSP samples displayed a dominating amorphous halo, as depicted in Figure 5a. This halo had a broad peak at 2θ = 20°, which was a typical XRD peak of xyloglucan. The intensity of the peak gradually decreased as the temperature increased [31]. Figure 5b shows the XRD curves of each PS. It is generally believed that amylopectin is the primary component of the crystalline region of starch, while amylose is found in the amorphous region [34,35]. It can be seen from the XRD curves of each PS in Figure 5b that the native PS showed strong diffraction peaks at 2θ values of 5.6°, 17°, 22°, and 24°, indicating that starch granules had traditional B-type crystalline patterns [36]. Similar findings were reported for potato starch by Kaul et al. [37]. PS-50 displayed peaks at 2θ of 17°, 22°, and 24°; however, the intensity of the peaks at 17° and 22° had decreased, indicating that crystallinity had been lost. PS-70 and PS-90 displayed similar peaks but with reduced intensity in all four peaks—some of which even disappeared—and other diffraction peaks decreased. Moreover, the degree of crystallinity index of PS, PS-50, PS-60, PS-70, and PS-90 was quantitatively estimated to be 24.6%, 17.9%, 16.6%, 13.2%, and 12.5%, respectively. The XRD results indicate that the higher the pretreatment temperature, the greater the damage to the crystal/amorphous structure of TSP and PS.

## 3. Discussion

The HPSEC results show that TSP was mainly composed of glucose (Glc, 52.1%), xylose (Xyl, 29.7%), and galactose (Gal, 18.2%). The literature [15,16] confirmed that TSP is a xyloglucan structure, consisting of a backbone with (1→4) -β-D-glucan that is partially substituted with α-D-xylopyranose at the O–6 position. Some of these xylose residues are replaced at the O–2 position with (1→2) β-D-galactosyl units; however, starch primarily includes amylose and amylopectin in a certain proportion, which are composed of glucose units connected by α-1, 4 and α-1, 6 glycosidic bonds [38,39,40]. The presence of side chains-xylose and two ring xylose–galactose combinations have a major effect on the solubility and hydration of TSP [2].

The structure of polysaccharides and their characteristics are directly correlated [2]. TSP contains three types of sugars viz. Glu, Xyl, and Gal with a molar ratio of 2.86:1.63:1, whereas starch is only composed of glucose units. The RVA result and rheological study indicate that TSP had no obvious PT compared to PS gelatinization, as its viscosity or *G*′ and *G*″ both gradually increased from the start of heating. It is speculated that the significant difference in pasting behavior and rheological results between TSP and PS might be due to the fact that the galactose units of TSP are more hydrophilic than the glucose unit of PS.

The SEM results indicate that as the temperature increased, the morphology of TSP particles transformed from an irregular lump shape to a leaf-like structure, followed by thinner flakes, but the size of the TSP flakes did not change much. It is speculated that the TSP particles, due to their very high concentration, relatively high molar mass, and poor water solubility, contributed to the slow dissolution or dispersion of the TSP into the aqueous solution, leading to the viscosity in the pasting behavior and the *G*′ and *G*″ in the rheological properties of TSP increasing during the whole heating process. Moreover, TSP kept its high viscosity almost unchanged in the RVA results, indicating that it had excellent stability at high temperature (95 °C). These results are consistent with the molecular parameters and microstructure. That is, the values of *M_w_* were slightly decreased from 9.622 × 10^5^ g/mol of TSP-50 to 8.584 × 10^5^ g/mol of TSP-90, indicating the relative stability of its structure. The study of crystallization characteristics showed that the amorphous properties of TSP-50 and TSP-90 had relatively small changes compared to native TSP. However, based on the SEM morphology, it is speculated that the starch granules in morphology undergo two obvious stages: first absorbing water and expanding, followed by disrupting and dissolving in aqueous solution. In the first stage, the viscosity and *G*′ and *G*″ of PS remained almost constant, followed by increasing sharply and achieving a maximum. In the second stage, the breakage of PS granules in suspension at high temperature occurred, resulting in a decrease in peak viscosity during the pasting behavior.

Above all, the findings show that the effects of treatment temperature on the morphological characteristics, pasting behavior, rheological properties, and microstructure of TSP samples were different from those of PS granules. This difference might be based on the different dispersion mechanisms of TSP and PS. Moreover, our results are highly consistent with the report on the dispersion mechanisms of TSP and starch [10]. TSP possibly undergoes a straightforward dispersion and then dissolves in aqueous solution during the heating process, while PS undergoes an initial expanding of particles followed by disintegration and dispersion.

## 4. Materials and Methods

### 4.1. Materials

TSP of food grade was purchased from Henan Luole Food Additives Co., Ltd. (Zhengzhou, China), which was supplied by Bhansali Agro Industries Pvt. Ltd. (Sangali, India) (viscosity of 4% paste by Brooke Field Viscometer Spidle No.4, at 20 RPM at 25 °C, around 6810 CPS). PS was obtained from Hangzhou Starpro Co., Ltd. (Hangzhou, China). All other reagents used in this study were analytical grade. Ultra-pure water was used to prepare samples throughout the experiments.

### 4.2. Preparation of TSP and PS at Different Temperatures

TSP and PS (0.5 g) were weighed and dispersed in 100 mL water. The dispersed solutions were placed on a magnetic stirrer at 50, 60, 70, and 90 °C for 30 min. After cooling to room temperature, half of the dispersion was centrifuged and precipitated with ethanol, followed by freeze-drying for structural features (HPSEC-PAD, SEC-MALLS, XRD, FT-IR) testing, and the other half of the dispersion was directly freeze-dried to observe the morphological changes under different temperature treatments (SEM). After drying, the obtained fractions were put into a dryer and labeled TSP-50, TSP-60, TSP-70, TSP-90, PS-50, PS-60, PS-70, and PS-90, respectively. It should be noted that TSP and PS could disperse well at the above temperatures, but might not have completely dissolved in aqueous solution.

### 4.3. HPSEC and SEC-MALLS Determination

High-performance anion-exchange chromatography (HPAEC-PAD) was used to determine the monosaccharides of TSP (untreated and extracted at different temperatures). Samples of 10 mg were hydrolyzed with 10 mL 4M trifluoroacetic acid (TFA) for 2 h at 121 °C and then diluted (×10) with ultrapure water [41]. The monosaccharide analysis was performed using a Dionex ICS-5000^+^ system (Thermo Fisher Scientific, Waltham, MA, USA) equipped with a pulsed amperometric detector (HPAEC-PAD) and a Carbo Pac^TM^ PA10 column (2.0 × 250 mm, Thermo Fisher Scientific, Waltham, MA, USA). Separation and quantitative analysis of monosaccharides were carried out in TSP using the gradient elution method. Conditions of leaching solution: 0–30 min, 13.5% 200 mmol/L NaOH, 86.5% ultrapure water; 30~60 min, 13.5% 200 mmol/L NaOH, 75% 200 mmol/L NaAc, 11 5% ultrapure water; 0.5 mL/min. Column temperature: 28 °C. Injection volume 25 μL. Standard monosaccharides such as D-glucose (Glc), D-galactose (Gal), D-xylose (Xyl), and arabinose (Ara) were used for calibration.

Using the method described by [42], the molecular characteristics of TSP fractions isolated at various temperatures were also determined. TSP-50, TSP-60, TSP-70, and TSP-90 were dissolved directly in mobile phase under magnetic stirring overnight (0.1 M NaNO_3_ and 0.02% (*w/w*, NaN_3_) with a concentration of 1.0 mg/mL at room temperature (25 °C). Before analysis, the sample solutions were centrifuged and passed through a 0.22 μm membrane filter, and 100 μL were injected into the chromatographic system. The molecular parameters of TSPs were determined on a size exclusion chromatograph equipped with multiple detectors (Wyatt SEC-MALS system, Wyatt Technology Santa Barbara, CA, USA). The SEC-MALLS system consists of a high-pressure injection system (Wyatt Technology, Santa Barbara, CA, USA), a degasser (GASTORR TG-14, GenTech Scientific Inc., Westbury, NY, USA), and a pump (S-1500, SSI, Cincinnati, OH USA). The SEC column (TSK-gel 3000 SWXL column, 7.8 × 300 mm, Tosoh Bioscience LLC, San Francisco, CA, USA) was kept at room temperature and protected by an OHpak SB-G guard column (Shodex, Tokyo, Japan). Using standard dextran (Mw = 41,400 Da), the detectors were normalized and the inter-detector volume was calculated. The ASTRA program, version 5.3.4 (Wyatt Technology), was used to carry out data collection and calculations.

### 4.4. Rapid Visco Analyser (RVA)

As per the description of Dos Santos et al. [38], with some modification, the pasting profiles of TSP and PS with different concentrations were characterized using RVA (Perten Instruments, Warriewood, Australia). Before the RVA testing, a stirrer equipped with RVA was used to thoroughly and quickly stir the TSP dispersion until the sample was fully dispersed. Briefly, the sample (2.5 g or 3.0 g, dry basis) was dispersed in an aluminum tube with ultra-pure water (25.5 g or 25.0 g, respectively) and the dispersion comprised 28.0 g (labeled TSP-2.5, TSP-3.0, PS-2.5, and PS-3.0, respectively). The programs were named Standard 1 and Standard 2, and the viscosity was expressed as a function of time, with cP as the unit. Then, the sample was pre-heated to 50 °C, then heated to 95 °C at different rates and cooled to 50 °C after being held at 95 °C for a period of time. The shear viscosity of the sample during these processes was measured at 960 r/min and 160 r/min. The detailed steps of measurement program Standard 1 and Standard 2 are shown in Table S2. The pasting parameters, including the pasting temperature (PT), peak viscosity (PV), peak time, minimum viscosity (MV), breakdown (BD = PV − MV), final viscosity (FV), and setback (SB = FV − MV), were characterized. In addition, the stability of TSP-3.0 and PS-3.0 was examined for 30 min at two distinct temperatures (70 °C and 95 °C).

### 4.5. Rheological Measurements

The rheological measurements of TSP and PS pastes were characterized according to the description of Chen et al. [23], with some modifications. Briefly, TSP (20%, *w/w*) and PS dispersions (20%, *w/w*) were characterized using a rotational rheometer (Haake Mars60, Thermo Scientific, Waltham, MA, USA) with a parallel plate configuration (P35 TiL) with a fixed gap of 1 mm. The samples of TSP and PS were both stirred magnetically for 30 min at room temperature (25 °C) to ensure full and uniform dispersion before rheological testing. For temperature sweep measurements, dispersions were heated from 40 °C to 90 °C and then cooled from 90 °C to 25 °C, with each step occurring at a rate of 2 °C/min. During this heating and cooling process, small-amplitude oscillatory shear tests were performed. The shear strain was set at 2% and the frequency was 1 Hz (this condition was confirmed to be within the linear viscoelastic regime; the strain sweeps are shown in Figure S3). The frequency sweep was measured as ranging from 0.1 to 40 Hz at 25 °C with the strain amplitude controlled at 2%. To reduce the amount of water lost during the test, a thin film of silicone oil was applied to the sample edge.

### 4.6. Microstructure Characterization of TSP and PS at Different Temperatures

Scanning electron microscopy (SEM, JSM-6390LV, Tokyo, Japan) was used to observe the microstructure of native TSP, PS, and samples treated at different temperatures. Double-sided adhesive tape was used to secure the samples to the support prior to observation. The vacuum sputtering instrument was gold-plated for 60 s at 15 mA. Every sample was observed at 20 kV.

Using X-ray diffraction (XRD, D8 Advance, Bruker Co., Ettlingen, Germany) with Cu-Kα radiation (λ = 1.54 Å), an accelerating voltage of 40 kV, and a tube current of 40 mA, the crystalline structure of all samples was recorded. The measurements were performed with an angular interval varying from 5° to 40° (2θ range), with 5° per minute for scanning, a 0.02° scan step, and 0.4 s per step.

All samples were determined by Fourier transform infrared (FT-IR) spectroscopy on a spectrometer (Alpha, Bruker Optics Ltd., Ettlingen, Germany) using the potassium bromide (KBr) pellet methods in dry air. After thoroughly mixing, the samples and KBr were pressed into tablets in a ratio of 1:200. At a resolution of 4 cm^−1^, the spectra were scanned between 4000 and 500 cm^−1^.

## 5. Conclusions

In this work, we investigated the changes in the structure and characteristics of TSP under different temperature conditions. It was found that TSP was mainly composed of glucose, xylose, and galactose in a ratio of 2.86:1.63:1. The galactose and xylose distributed in the side chain of TSP had a significant impact on the hydration and solubility of TSP, which in turn greatly influenced the pasting behavior and dynamic rheological properties. The pretreatment did not change the chemical structure of TSP or PS at different temperatures but did disrupt their crystal structure or weakened their peak intensity. It was shown that TSP did not exhibit a traditional pasting curve like starch, but had a higher peak viscosity and stronger thermal stability than PS. The dynamic rheology study clearly showed that *G*′ and *G*″ values of TSP were much greater than those of PS, exhibiting good viscoelasticity behavior and high-temperature resistance characteristics. The morphological differences between TSP and PS under different treatment temperature were observed by SEM. In this work, we compared the differences in rheological, thermal, and structural properties between TSP and PS, and did not study the effect of TSP on PS gelatinization, as TSP can protect PS by endowing it with thermal stability, thereby inhibiting starch gelatinization to a certain extent.

## Figures and Tables

**Table 1:** Experimental data of molecular weight parameters and monosaccharide ratios of TSP at different treatment temperatures.

Sample	*M*_w_ × 10^5^ (g/mol)	*M*_n_ × 10^5^ (g/mol)	*M*_w_ */M*_n_	*R*_n_ (nm)	*R*_w_ (nm)	Glc:Xyl:Gal
TSP-50	9.622	9.118	1.055	70.1	70.9	2.36:1.35:1
TSP-60	9.381	8.938	1.050	75.1	76.3	2.66:1.52:1
TSP-70	9.183	8.763	1.048	75.8	77.1	2.80:1.62:1
TSP-90	8.584	8.191	1.048	73.1	74.5	2.89:1.70:1

Note: *M*_w_, *M*_n_: weight average molecular weight, number average molecular weight. *R*_n_, *R*_w_: number average radius of gyration, weight average radius of gyration.

**Table 2:** Pasting parameters of TSP and PS (Standard 1).

Samples	PT (°C)	PV (cP)	Peak Time (min)	BD (cP)	SB (cP)	FV (cP)
PS-2.5	69.90 ± 0.57	4560 ± 16	3.70 ± 0.09	2687 ± 31	257 ± 21	2214 ± 6
PS-3.0	69.43 ± 0.04	5933 ± 194	4.10 ± 0.14	3576 ± 190	573 ± 84	2930 ± 88
TSP-2.5	ND	6038 ± 46	5.03 ± 0.23	567 ± 36	2523 ± 46	7995 ± 237
TSP-3.0	ND	8922 ± 680	5.13 ± 0.33	639 ± 151	2944 ± 221	11,227 ± 751

Note: PT, PV, BD, SB, and FV represents pasting temperature, peak viscosity, breakdown, setback, and final viscosity, respectively. Data are means ± SD, ND: not detected.

## Data Availability

Data are contained within the article.

## Supplementary Materials

The following supporting information can be downloaded at: https://www.mdpi.com/article/10.3390/molecules29112622/s1, Figure S1: The elution profiles of TSP at different temperatures detected by SEC-MALS; Figure S2: The second cycle of heating and cooling process using mars 60. Figure S3: The strain sweeps (0.01–100%) of TSP and PS; Table S1: Pasting parameters of TSP and PS (Standard 2); Table S2: Steps of Standard 1 and Standard 2.
