# Supplementary material for: Influence of Temperatures on Physicochemical Properties and Structural Features of Tamarind Seed Polysaccharide
Source: Molecules. 2024 Jun 3;29(11):2622. doi: 10.3390/molecules29112622 (PMC11174022; doi:10.3390/molecules29112622)
Supplement: Supplementary file 1 [file molecules-29-02622-s001.zip › molecules-2973734-supplementary.pdf]

molecules

MDPI

Supplementary Materials

Influence of Temperatures on Physicochemical Properties and Structural Features of Tamarind Seed Polysaccharide

Figure S1. The elution profiles of TSP at different temperatures detected by SEC-MALS.

Figure S2. The second cycle of heating and cooling process using mars 60

Figure S3. The strain sweeps (0.01–100%) of TSP and PS.

Table S1. Pasting parameters of TSP and PS (Standard 2).

Table S2. Steps of Standard 1 and Standard 2.

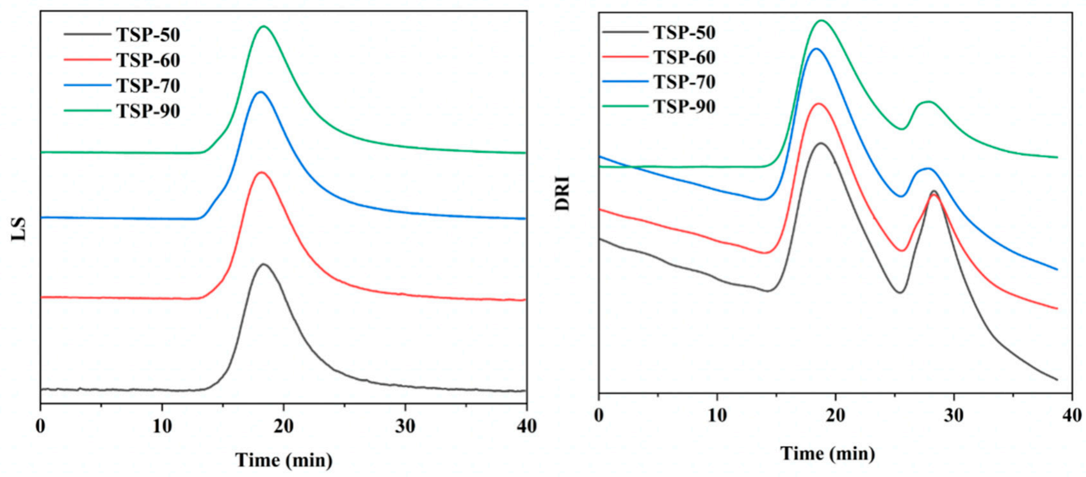

Figure S1. The elution profiles (LS, DRI) of TSP at different temperatures detected by SEC-MALS.

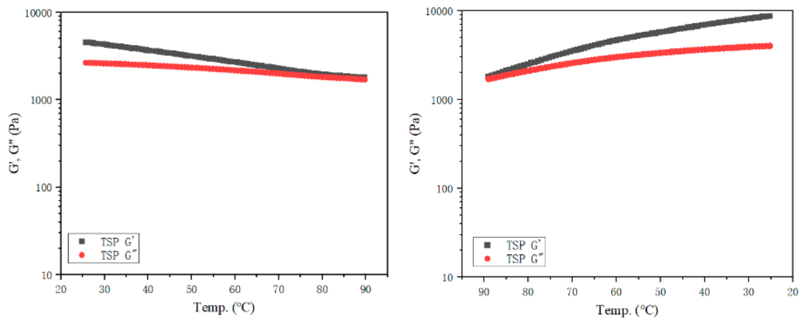

Figure 2. The second cycle of heating and cooling process of TSP using mars 60.

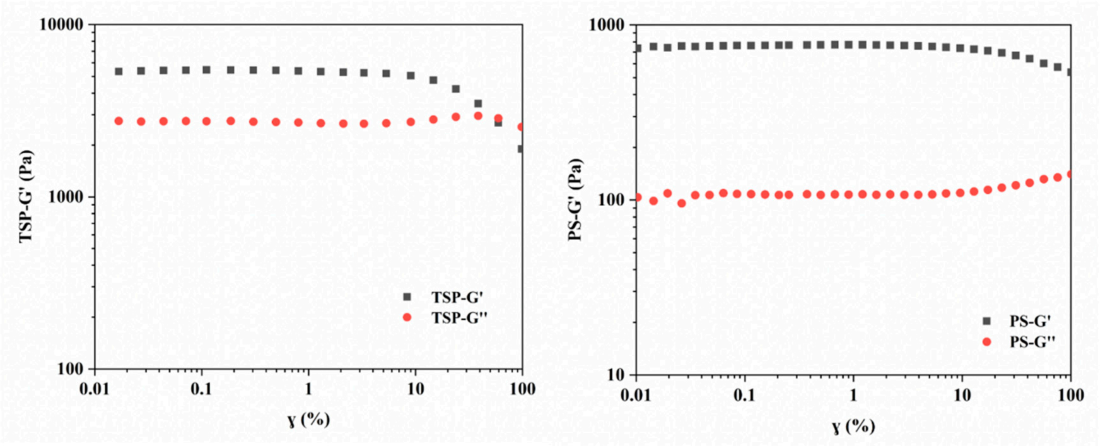

Figure S3. The strain sweeps (0.01-100%) of TSP and PS.

Table S1. Pasting parameters of TSP and PS (Standard 2).

Sample	PT (°C)	PV (cP)	Peak Time (min)	BD (cP)	SB (cP)	FV (cP)
PS-2.5	64.13±0.04	4382±6	8.80±0.00	3049±19	443±29	1775±5
PS-3.0	63.73±0.04	5229±141	10.27±0.47	3613±109	555±13	2210±45
TSP-2.5	ND	6430±428	13.47±1.51	792±54	3178±104	8848±677
TSP-3.0	ND	9485±320	12.67±0.38	1193±150	4033±516	12325±986

Table S2. Steps of Standard 1 and Standard 2.

step	Temp. or Speed	Time (h: min: s)	
		Standard 1	Standard 2
1	50 °C, 960 r/min	00: 00: 00	00: 00: 00
2	50 °C, 160r/min	00: 00: 10	00: 00: 10
3	50 °C, 160r/min	00: 01: 00	00: 01: 00
4	95 °C, 160r/min	00: 04: 42	00: 16: 00
5	95 °C, 160r/min	00: 07: 12	00: 26: 00
6	50 °C, 160r/min	00: 11: 00	00: 41: 00
end		00: 13: 00	00: 43: 00
